# A method for achieving complete microbial genomes and improving bins from metagenomics data

**DOI:** 10.1371/journal.pcbi.1008972

**Published:** 2021-05-07

**Authors:** Lauren M. Lui, Torben N. Nielsen, Adam P. Arkin

**Affiliations:** 1 Environmental Genomics and Systems Biology Division, Lawrence Berkeley National Laboratory, Berkeley, CA, United States of America; 2 Department of Bioengineering, University of California, Berkeley, California, United States of America; 3 Innovative Genomics Institute, Berkeley, CA, United States of America; DAL, CANADA

## Abstract

Metagenomics facilitates the study of the genetic information from uncultured microbes and complex microbial communities. Assembling complete genomes from metagenomics data is difficult because most samples have high organismal complexity and strain diversity. Some studies have attempted to extract complete bacterial, archaeal, and viral genomes and often focus on species with circular genomes so they can help confirm completeness with circularity. However, less than 100 circularized bacterial and archaeal genomes have been assembled and published from metagenomics data despite the thousands of datasets that are available. Circularized genomes are important for (1) building a reference collection as scaffolds for future assemblies, (2) providing complete gene content of a genome, (3) confirming little or no contamination of a genome, (4) studying the genomic context and synteny of genes, and (5) linking protein coding genes to ribosomal RNA genes to aid metabolic inference in 16S rRNA gene sequencing studies. We developed a semi-automated method called Jorg to help circularize small bacterial, archaeal, and viral genomes using iterative assembly, binning, and read mapping. In addition, this method exposes potential misassemblies from k-mer based assemblies. We chose species of the Candidate Phyla Radiation (CPR) to focus our initial efforts because they have small genomes and are only known to have one ribosomal RNA operon. In addition to 34 circular CPR genomes, we present one circular Margulisbacteria genome, one circular Chloroflexi genome, and two circular megaphage genomes from 19 public and published datasets. We demonstrate findings that would likely be difficult without circularizing genomes, including that ribosomal genes are likely not operonic in the majority of CPR, and that some CPR harbor diverged forms of RNase P RNA. Code and a tutorial for this method is available at https://github.com/lmlui/Jorg and is available on the DOE Systems Biology KnowledgeBase as a beta app.

## Author summary

Since we cannot culture many microorganisms that are found in the environment, animals, and the human body, scientists rely on shotgun metagenomics to reveal their genomes and to infer their traits and capabilities. However, shotgun metagenomics often only provides fragmented genomes due to limitations of available sequencing technology and bioinformatics tools. We present a semi-automated method called Jorg that can be used to improve and eventually complete (*i*.*e*., circular with no misassemblies) prokaryotic and viral genomes from short read metagenomics data, and also include quality checks for misassemblies and completeness. As a proof-of-concept we circularized 36 bacterial genomes and two megaphage genomes. For comparison, there are only ~100 known circularized bacterial genomes from metagenomes from ~30 other studies. We also demonstrate findings that illustrate the utility of circularizing genomes by discovering new biological patterns in Candidate Phyla Radiation species. High-quality circularized genomes produced using this tool also can be used as scaffolds to improve future genome assemblies and as data to improve identification of species in microbiomes.

## Introduction

Shotgun metagenomics and marker gene sequencing are powerful tools to survey and study organisms that we cannot yet isolate and culture in the laboratory. This is especially true for environmental samples where culturability estimates for bacterial and archaeal communities range from ~22–53% for soil, ~10–70% for ocean and lakes, and ~8–32% for ocean sediment [1]. Scientists have turned to shotgun metagenomics to provide genome-resolved analysis of complex samples, but assembling genomes from shotgun metagenomics data is inherently more difficult than assembling those from cultured isolates. Challenges in metagenomics assembly arise from the heterogeneity of samples, available sequencing technology, and the limitations of bioinformatics algorithms we use for assembly and genome binning [2]. Metagenomes contain uneven amounts of an unknown number of genomes, which creates a compounded computational problem in terms of simplifying assumptions, time, and computer memory.

In the 1990s when the first genomes were sequenced and assembled, scientists used long reads from Sanger sequencing and overlap layout consensus (OLC) methods for assembly [3]. With the development of next-generation sequencing technologies, we gained the ability to sequence millions of reads at a massively reduced cost, but using traditional OLC algorithms became too computationally intensive. The computational complexity of OLC algorithms scale as the square of the number of input reads (because each read is compared to every other read), so they are impractical for datasets of millions of reads, compared to the thousands of reads generated from Sanger sequencing. More specifically, overlap identification is typically determined by dynamic programming, which scales by the square of the number of reads (*d*) and their length (*n*), so the complexity is *O*(*d*^*2*^*n*^*2*^) or *O*(*N*^*2*^). Most OLC assemblers combine dynamic programming with suffix tree algorithms which are *O*(*N+α*), where *α* is the total number of overlaps and thus have complexity of *O*(*d*^*2*^). To handle the deluge of sequencing data (in terms of the volume of reads and projects) de Bruijn graph assembly methods were developed.

The time and memory complexity of de Bruijn based assembly algorithms typically scale with the size and complexity of the metagenome instead of the number of reads [4]. The de Bruijn graph approach decomposes reads into k-mers, or short subsequences of length *k*, and only unique k-mers add nodes to the graph [4]. This reduces computational requirements compared to OLC assemblers, but can also introduce misassemblies. Due to the decomposition of reads into k-mers, context is lost and it is possible for the graphs to contain paths that do not correspond to real genomic sequence [4,5] (although it is possible to recover some of the context, such as by mapping input reads back to the graph [4]). Traditional OLC assemblers such as the Celera Assembler [6], SGA [7] and MIRA [8] ensure that only contigs consistent with actual genome sequence are produced (this is sometimes referred to as maintaining read coherence). This is not to say that OLC assemblers do not have misassemblies [9], but some of the valuable context within the original reads may be lost using k-mer based assembly approaches.

Using long-read sequencing can help overcome some of the issues with k-mer based assembly and newer assemblers for this type of data have started using OLC assembly methods again [5]. However, long-read sequencing requires much larger amounts of DNA (micrograms) that is high quality and high molecular weight (10-50kb) as compared to short read technologies (as little as 1 nanogram) [10,11]. Extracting enough high molecular weight DNA can be limited by sampling costs and available biomass, especially for certain types of environmental samples, so short read sequencing may sometimes be the only option.

Beyond assembly, a key challenge with metagenomics is grouping contigs into genome bins. We use “contig” in the way it was originally defined by Rodger Staden, where a contig is a set of overlapping segments of DNA from shotgun sequencing [12]. It is rare for a complete genome to be assembled into a single piece *de novo* from short reads, so contigs are grouped into “bins,” often based on coverage and tetranucleotide frequencies. If two contigs belong to the same genome, they are expected to have similar coverage and tetranucleotide profiles [13]. However, coverage has problems for multiple reasons. If a particular microbe is growing rapidly, some regions may have higher coverage than the rest of the genome [14]. In addition, for organisms where the copy number of ribosomal RNA (rRNA) operons exceeds unity, the contig(s) with the rRNA genes will not have the same coverage as the rest of the contigs in the genome. This is also true of other multi-copy genes and other repetitive elements. Tetranucleotide frequencies are problematic because horizontally transferred regions may have different frequencies than the rest of the genome [15] and this can result in such pieces being put into different bins by the binning algorithm. Despite these issues, binning is helpful in identifying potential genomes in metagenomics data, especially when using short read sequencing technologies.

To evaluate the quality of a bin, the metrics of “contamination” and “completeness” are often used. Completeness and contamination are detected generally by looking for violations of conserved features of complete isolate genomes. Such features include having complete sets of universally (or at least phylogenetically) conserved single-copy protein genes without any duplication or excessive variation in tetranucleotide frequency. Other measures of completeness have been suggested, such as establishment of a core conserved set of ubiquitous genes. Tools such as RefineM [16] and CheckM [17] apply these rules to assemblies to determine completeness and contamination for bacterial and archaeal genomes. However, these tools are not always accurate for species that are not well studied. Candidate Phyla Radiation (CPR) species are often classified as having 60–80% completeness by these tools, even for circular genomes of these species [18]. To overcome challenges of binning, scientists have started to assemble circular, complete genomes from metagenomes [18–27], which are also called CMAGs (complete metagenomic-assembled genomes) [28]. In comparison to genome bins, a high quality reference collection that controls for misassemblies and is composed of circular genomes (1) provides more accurate inference of identity and estimation of capabilities of uncultured microbes within complex microbiomes, (2) allows more accurate taxonomic assessment of the composition of these microbiomes through better linkage of marker genes in single organisms, (3) provides high-quality scaffolds on which reads can be assembled, both to allow measures of strain variation within a microbiome study and to aid in better assembly of reads across many microbiome samples, and (4) affords the ability to study synteny and genomic context of genes in these organisms. In addition, while there are existing methods for generating high-quality MAGs, there is evidence that these MAGs still contain significant contamination by exogenous sequence and have misassemblies triggered by lack of read coherence [29]. Circularization of genomes helps increase the likelihood that that there is little to no contamination in the assemblies. Despite the advantages of circularizing genomes, very few metagenomics studies to date (<30) have published circular genomes [18,19,28,20–27].

We describe a semi-automated method called Jorg that facilitates recovery of circular archaeal, bacterial, and viral genomes from metagenomics data and that also provides checks for misassemblies. To facilitate this method, we have developed scripts and a tutorial that are available on GitHub (https://github.com/lmlui/Jorg), as well as a DOE Systems Biology Knowledge Base (KBase) app that is currently in beta (https://appdev.kbase.us/#appcatalog/app/kb_jorg/run_kb_jorg/beta) [30]. This method is not intended to help assemble complete eukaryotic microbial genomes, such as yeast, but may be used to help extend contigs in these species. It is also meant to be primarily used for small genomes with few repeats, but can be used to help extend contigs in all metagenome bins. In this study we do not focus on how many circularized genomes we can get from a dataset, but rather the method itself to help circularize bins of interest and to ensure that they are high quality. To assist with the travails of circularizing genomes, our method overcomes issues from using k-mer based assembly and automates iterative extension of contigs. Our general approach is to produce a “standard” metagenomic assembly, bin using a “standard” binning tool, extract reads based on k-mer similarity and reassemble these using a “standard” isolate-focused assembler. To demonstrate this method, we have obtained 34 circular CPR genomes, one circular Margulisbacteria genome, one Chloroflexi genome, and two circular megaphage genomes from 19 public and published metagenomics datasets. To our knowledge, only 41 other CPR circularized genomes have been published from 9 studies [18–26], so we believe this to be the largest presentation of circularized CPR genomes in a single study. With this set, we demonstrate findings that would likely be difficult without a large number of unique circularized genomes, including that ribosomal genes are likely not operonic in the majority of CPR and finding diverged forms of RNase P RNA in CPR species.

## Results

### Circularization method

To have confidence that the genomes we generate match real organisms, we looked for criteria that would indicate that a genome is circular and complete. The literature is replete with techniques and proposals for measuring the completeness of genomes and to what level they are complete [17,20], but these often have difficulty when encountering novel genomes because their criteria are based on known isolate genomes. We focus on evidence for incompleteness in terms of missing essential genes that are found across the tree of life. That is, we are more concerned with ensuring that anything we label a circularized genome meets basic criteria that indicates that it is not incomplete. We posit that a complete, circularized genome must satisfy at least the following conditions:

1. The genome is either circular or there is solid evidence that it is linear. While rare, linear bacterial genomes exist [31]. To represent a circular genome, a contig must have an exact overlap of the ends that is longer than any repeat in the genome. Linear genomes and chromosomes can be confirmed by finding paired-end reads that point inward from the ends of the contigs. If a genome is circular, there will be some paired-end reads that have incorrect orientation at the ends of the contigs, *i*.*e*., one read pointing outward on one end of the contig with the second read on the opposite end pointing outwards. Circularity can also be inferred by overlap at the ends of the contig (discussed further later in this section).
2. The genome has a full complement of rRNAs (16S, 23S, 5S), transfer RNAs (all amino acids represented), and RNase P RNA (since this is nearly universally necessary to process tRNA transcripts). Absence of any of these genes must be explained. We advocate using these as a check for a complete genome instead of single copy marker protein genes because checks for single copy marker protein genes can vary by clade; in only rare instances would these noncoding RNA genes be missing [32,33].
3. There is significant read coverage across the entire genome. Assemblies that rely on single reads for continuity are prone to error. With some exceptions for very high coverage organisms, we generally require minimum coverage no lower than 30% of the average coverage (typically >30X except in rare cases).

To develop and test this method, we mined the Sequence Read Archive (SRA) hosted at the National Center for Biotechnology Information (NCBI) [34] for metagenomic sequencing data generated from groundwater samples, where CPR are prevalent (see Table 1 for a list of the datasets used in this study). We focused on assembling CPR genomes because they are (1) small and thus easier to assemble, (2) to the best of our knowledge only have one set of rRNA genes. These two criteria gave us the easiest targets for circularization.

**Table 1:** Description of metagenomes in this study.

Identifiers	Study Description	Reference
ERX2165959	Groundwater from monitoring wells from naphthalene contaminated surface sediments, where effluent from the coal-tar contaminated groundwater surfaces	[35]
SRX1085364	Terrestrial subsurface C, N, S and H cycles cross-linked by metabolic handoffs	[20]
SRX1775573 SRX1775577 SRX1775579	Potential for microbial H2 and metal transformations associated with novel bacteria and archaea in deep terrestrial subsurface sediments	[25]
SRX1990955	Groundwater microbial communities from Rifle, Colorado—Rifle Oxygen_injection A2 metagenome	[20]
SRX2200098	Trichloroethene-dechlorinating enrichments of contaminated groundwater	[36]
SRX2838984	Coupling Microbial Communities to Carbon and Contaminant Biogeochemistry in the Groundwater-Surface Water Interaction Zone	[37–39]
SRX3024504 SRX3024507 SRX3024508	DNA from groundwater after nitrate injection, filter size 0.2μm and 0.1μm	[40]
SRX3307784	Subsurface groundwater microbial communities from S. Glens Falls, New York, USA—GMW37 contaminated, 5.8 m metagenome	[35]
SRX3348993	Development of a pipeline for high-throughput recovery of near-complete and complete microbial genomes from complex metagenomic datasets: Groundwater sample from aquifer—Crystal Geyser CG19_WC_8/21/14_NA	[41]
SRX3574179	Investigating microbial roles in methane emission, contaminant degradation, and biogeochemical cycles in an aquifer near a municipal landfill	Laura Hug Lab; https://uwaterloo.ca/hug-research-group/
SRX3602289 SRX3602720	Groundwater microbial communities from the Äspö Hard Rock Laboratory (HRL) deep subsurface site, Sweden	Mark Dopson Lab; https://lnu.se/en/staff/mark.dopson/
SURF_D	Groundwater samples from the Sanford Underground Research Facility (SURF)	[42]

We focused on groundwater datasets because they have a higher fraction of CPR. Many of the datasets are from studies of anthropologically contaminated sites. All identifiers are SRA except for SURF_D.

**Fig 1:**
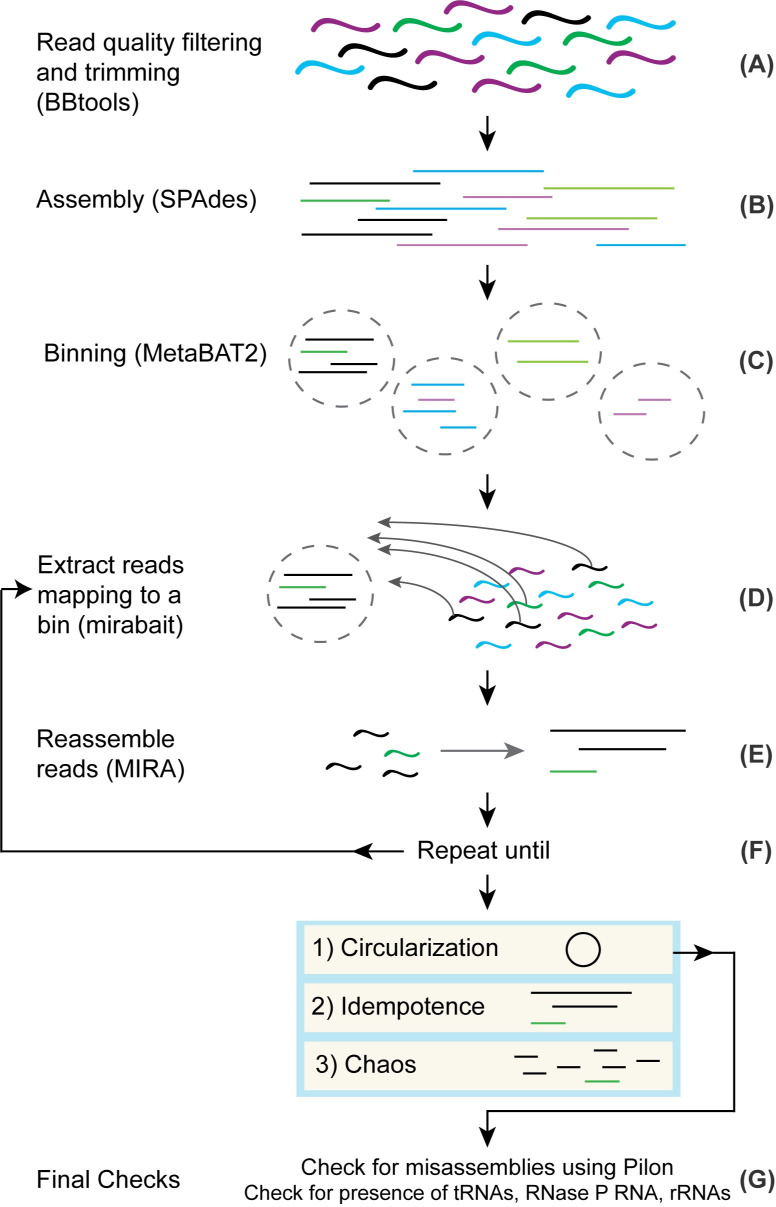
General method for circularizing genomes from metagenomes. (A) Reads have adapters trimmed and low-quality reads are filtered using BBtools. (B) Processed reads are assembled into contigs using SPAdes. (C) Contigs are grouped into bins using MetaBAT 2. (D) After choosing a bin for circularization, reads mapping to the bin are extracted from the original processed reads and used as input into (E) where they are assembled into contigs using MIRA. (F) Steps D and E are repeated as necessary until the bin is deemed to be in a Circularization, Idempotence, or Chaos state. (G) If a bin is deemed circular, we do final checks for misassemblies using Pilon and for the presence of rRNA, tRNAs, and RNase P RNA before officially calling the bin a circularized genome.

The first steps of the method are standard to regular metagenomics assembly pipelines. For each metagenome, we trimmed the reads to remove any remaining Illumina adapter fragments and low-quality ends, as well as whole reads that weren’t of sufficient quality, using BBtools (Fig 1A). Next, we assembled the processed reads using SPAdes [43,44] (Fig 1B). We proceeded with successful assemblies and used MetaBAT 2 [45] (Fig 1C) to produce a collection of bins for each. We went through 188 assembled metagenomes and picked bins with 5 or fewer contigs and coverage above 40X, although we made exceptions for bins that looked promising, such as a bin with many contigs, but with one or two large contigs that comprise most of the bin’s sequence length (Table 2). We used GTDB-Tk [46] to classify the bins and picked a set of CPR bins. We used these bins as “bait” to select read pairs for use with the isolate-focused assembler MIRA (Fig 1D).

**Table 2:** List of 36 circularized bacterial genomes in this study.

Genome ID	GTDB-TK Taxonomy (Class)	Genome Size (bp)	Original Bin Stats		
			Num of Contigs	Coverage	Size of Bin (bp)
ERX2165959_bin_184	Paceibacteria	523910	2	126X	531828
ERX2165959_bin_23	Microgenomatia	1147419	1	134X	1146985
ERX2165959_bin_53	Microgenomatia	646630	3	116X	780569
ERX2165959_bin_80	Microgenomatia	1014979	1	216X	1024366
SRX1085364_bin_95	Microgenomatia	819458	4	109X	826172
SRX1775573_bin_5	Gracilibacteria	998919	1	104X	999239
SRX1775577_bin_36	Gracilibacteria	999108	1	74X	998727
SRX1775579_bin_0	Dojkabacteria	732899	1	51X	733907
SRX1990955_bin_0	Margulisbacteria (phylum); WOR-1	1676518	1	56X	1673447
SRX1990959_bin_38	Paceibacteria	585024	2	35X	582583
SRX2200098_bin_18	Chloroflexota (phylum); Dehalococcoidia	1408204	1	2000X	1408334
SRX2838984_bin_5	Paceibacteria	1030062	7	274X	1030337
SRX3024504_bin_47	Paceibacteria	672946	1	41X	673298
SRX3024507_bin_14	ABY1	1064268	4	48X	1106392
SRX3024507_bin_96	Paceibacteria	672946	1	29X	673073
SRX3024508_bin_27	Paceibacteria	672946	1	91X	673184
SRX3307784_bin_186	Paceibacteria	581622	4	174X	578873
SRX3307784_bin_197	Paceibacteria	822324	8	203X	962091
SRX3307784_bin_224	Microgenomatia	646579	3	118X	780569
SRX3307784_bin_45	UBA1384	872881	1	93X	872947
SRX3307784_bin_80	Microgenomatia	1013439	1	220X	1024366
SRX3307784_bin_91	Paceibacteria;	523446	3	129X	531688
SRX3348993_bin_93	Saccharimonadia	1005778	1	64X	1005352
SRX3574179_bin_116	Saccharimonadia	949592	4	37X	1000785
SRX3574179_bin_12	ABY1	1027227	1	135X	1028393
SRX3574179_bin_242	Microgenomatia	1127729	3	48X	1130277
SRX3574179_bin_244	Paceibacteria	707009	1	106X	708095
SRX3574179_bin_38	Paceibacteria	682291	5	124X	680516
SRX3574179_bin_63	ABY1	1209998	2	88X	1209921
SRX3574179_bin_75	ABY1	954552	3	152X	979051
SRX3602289_bin_51	Microgenomatia	852061	2	94X	853126
SRX3602720_bin_127	Paceibacteria	750123	18	160X	826132
SRX3602720_bin_74	ABY1	1035066	3	32X	1041342
SRX5650846_bin_20	Microgenomatia	947771	9	53X	949620
SURF_D_bin_21	Microgenomatia	978848	3	193X	978553
SURF_D_bin_31	Microgenomatia	1496927	5	159X	1501138

Thirty-four of the genomes are CPR, one is a Margulisbacteria, and one is a Chloroflexi as classified by GTDB-Tk. The coverage, original number of contigs, and length of the original bin is also included.

The purpose for assembling contigs first with SPAdes and then switching to MIRA with a subset of reads is that the computational requirements of MIRA make it impractical as a metagenome assembler. This is in part because MIRA does full alignment of the reads during assembly. We would like to note that OLC metagenomics assemblers exist [5], but their memory and time requirements are high compared to SPAdes or are not appropriate for assembling bacterial genomes with paired-end sequencing data. MIRA has been used to extract mitochondrial genomes [47] from eukaryotic sequencing projects. Our approach is very similar; instead of providing seed sequences to separate the mitochondrial genomes from the eukaryotic DNA, we use bins as seeds to separate genomes from the entire metagenomics dataset. In our experience, MIRA produces superior results for isolates, and it also provides additional features that benefit our method. MIRA comes with the tool mirabait, which provides support for extracting read pairs based on k-mer content. MIRA also has a variety of features that help expose problematic parts of assemblies. For example, MIRA sets tags to indicate parts of the assembly that may require manual intervention, based on changes in coverage, GC, and other anomalies. These tags are extremely useful in conjunction with traditional assembly finishing tools such as Gap4/Gap5 [48].

Perhaps even more critical to this method than MIRA’s utilities is the fact that MIRA also ensures read coherence as an overlap-based assembler, unlike k-mer based assemblers like SPAdes. SPAdes is commonly used for metagenomics assembly, and in our experience, produces results that are as good as any other metagenomics assembler that is typically recommended [4]. However, there are often misassemblies caused by running SPAdes on a large and heterogeneous collection of metagenomes with the same set of k-mers. Ideally, the user would conduct tests to find the optimal collection of k-mers for each individual metagenome, but this step is time consuming. Thus, many users—us included—pick a canonical set like 21, 33, 55, 77, 99 and 127 that in most cases give the greatest contiguity in the assembly. Unfortunately, this practice can produce illusionary contiguity if the read coverage cannot support all of the k-mer sizes [5]. Larger k-mers increase contiguity, but the read coverage may not support them. By using MIRA, contigs that do not have read coherence may be exposed. It is possible to do the reassembly with a de Bruijn graph assembler like SPAdes, but in our experience, it does not do as well extending contigs because of the issue of read coherence. SPAdes assemblies often have issues at the ends of contigs, which often results in duplicates of contigs that are similar except at the ends.

After we used mirabait to extract read pairs that mapped to selected bins (Fig 1D) and reassembled them using MIRA (Fig 1E), we iterated these two steps (Fig 1F). This iterative process results in “digital primer walking” to extend the contigs of the bin, similar to primer or genome walking that was initially used to sequence genomes in the late 1980s to early 1990s [49]. At each iteration, reads with a portion mapping to any part of a contig will be included and can lead to extension or fusion of contigs. We specifically chose to reassemble all of the reads during each iteration to provide a more robust handling of repeats. On occasion, the extension of the contigs resulted in overlap with contigs from other bins and unbinned contigs. Manually including these contigs as part of the bait can speed up the process significantly. However, we also routinely examined intermediate results and, in some cases, we saw anomalous coverage values for different contigs indicating possible chimerism. If we saw the bin containing contigs with significantly different coverage values (>10% difference), we removed the offending contigs and restarted.

Read-baiting with mirabait includes reads that map to multiple bins, so we have additional checks at each iteration step to help remove contaminating contigs. We chose not to filter reads that have multiple mapping because (1) some genomes are spread across multiple bins, and (2) there are cases where conserved regions between genomes can’t resolve from unique mapping. After each iteration, we remove short contigs and filter contigs based on coverage to remove contaminating contigs.

We iterated read-baiting and assembly (Fig 1D–1F) until one of these outcomes occurred:

1. **Circularization.** For us to decide that this had occurred, we looked for a single contig with a significant—and exact—repeat at the ends. In addition, we required that the repeat be at least 100 nt in length, was longer than any other repeat in the contig, and did not match any of the other repeats.
2. **Idempotence.** In some cases, we observed no change in the assembled contigs after a round of read pair extraction and reassembly with MIRA. We examined some of these instances in detail and we believe the change in coverage causes MIRA to refuse to continue extending contigs. It is possible to adjust MIRA’s thresholds of what constitutes low and high coverage to allow contig extension to continue. However, this modification increases the risk of collapsing repeats or creating chimeric assemblies.
3. **Chaos.** There are cases where a bin is shattered into a multitude of pieces. We are not certain as to the exact cause, but this result is likely due to misassemblies from the initial SPAdes assembly (discussed in more depth in a later section). Chaos appears strongly correlated with GC and tends to occur more often when the GC content is high. We have investigated a few in more detail and for some found that the contigs that shatter have low 127-mer coverage as reported by SPAdes. We believe that Chaos bins are caused by lack of read coherence in the contigs and if that is indeed the case, there is little we can do. Once we see Chaos set in, it appears to be permanent.

After circularizing a contig, we did final checks for misassemblies with Pilon (Fig 1G). We used Pilon [50] on the contig and then we rotated it by half the length to ensure that the ends were in the middle and applied Pilon again (Fig 1G). We rotate the genomes because Pilon is not capable of covering the ends of a contig. While Pilon found minor insertions/deletions due to the circularization, it did not find any other issues in the genomes.

We next searched the genomes for a full complement of ribosomal RNAs (16S, 23S, 5S), tRNAs (all amino acids represented) and RNase P RNA to help check that the genome was correctly circularized and was not missing regions. For RNase P RNA, we needed to manually reduce score thresholds to find all RNase P RNAs (discussed in more detail in a later section). We were able to find tRNAs for all amino acids, although some tRNAs had Group I introns, making them difficult to detect. Structural RNAs are sometimes invaded by Group I introns, which is particularly true for tRNAs [Patricia Chan, private communication]. When a genome passed the final check with the detection of the set of non-coding RNAs, we considered the genome to be likely complete and circularized.

SRX3307784_bin_197 is an example of a bin that appeared to be circular, but did not pass the check of having RNase P RNA. The assembled contig had a solid 414 base pairs of overlap at the ends, ribosomal proteins present and tRNAs for all amino acids. However, we did not find a copy of RNase P RNA even when we lowered the detection threshold. This caused us to look closer and we discovered that there was another contig in the assembly which we had thought was contamination after the initial circularization. This contig has a copy of RNase P RNA and we were able to incorporate it into the assembly after we discovered a repeat that was too long for the reads to span and that Pilon did not detect. We came to the conclusion that this was a case of false circularization. To address the misassembly, we put the bin through more iterations with mirabait and MIRA, which resulted in a larger genome which passed all of the final checks.

To confirm that we had not inadvertently created chimeras during the circularization process, we did a few more checks on the genomes. We confirmed that there was the expected number of ribosomal RNA genes and length of genome for the taxonomic classification. In this case, all of the genomes are expected to have one ribosomal operon and a genome length between 0.5–1.5Mb [18,21]. We also confirmed the GTDB-tk taxonomy with SILVA taxonomy of the 16S rRNA genes if possible (S1 Table). As we previously mentioned, CheckM has not been trained on many Candidate Phyla Radiation genomes and thus is not an appropriate check of completeness and contamination for these genomes (the genomes in this study had a CheckM completeness of between 48–88%, except for the Chloroflexi genome which had a completeness of 99%), although CheckM indicated very little if any contamination (twenty-nine of the genomes had 0% contamination and the remaining seven had less than 3% contamination). We also checked genomes for even coverage (S1 Fig). Since CheckM does not have an appropriate database for CPR genomes, we used GUNC [51] as an additional check for chimerism. All of the circularized genomes passed the GUNC evaluation (S2 Table). With these additional checks, we concluded that our genomes were very likely assembled properly and were not chimeric.

The computational requirements for running Jorg depend on the number of reads and the initial size of the bin (S2 Fig). A bin that is approximately 0.5Mbp will typically take 1–2 hours for one iteration of Jorg. For example, one iteration of Jorg for SRX3307784_bin_186 took 52.5 minutes, on a machine with dual CPUs with Intel(R) Xeon(R) CPU E5-2620 v4 @ 2.10GHz processors and 256 GB of memory, for a bin that was initially 578873bp and had a reads file size of 22Gb. The baiting step time will be proportional to the size of the data, but assembly with MIRA will be proportional to the size of the bin and will typically dominate the iteration time as the initial bin size increases (S2 Fig).

In total we attempted to circularize 234 bins, so approximately 16% of the time we succeeded in creating a circularized genome out of a bin. However, our selection of bins was not random as we were heavily biased in favor of bins that we judged easiest to circularize, such as selecting bins classified as CPR, had relatively few contigs, and had solid coverage. We are confident that we can assemble more genomes from these datasets because we have also been able to circularize genomes from archaea and other bacteria; these genomes will be published in future papers. We intend to make these genomes generally accessible as we finish them.

In general, we do not recommend applying Jorg to large genomes (>4Mb) that likely have repeats longer than the library fragment size or to bins with large amounts of contamination. Genomes with long repeats will not be able be circularized by MIRA. Applying Jorg to the ZymoBIOMICS mock community that includes 8 bacteria and 2 yeast did not yield any circular genomes, as the members of the mock community are relatively large genomes that have significant repeats, including multiple rRNA copies (see Methods). However, we tested the ability to recover CPR genomes by including reads that mapped back to one of the genomes we circularized in this study (SRX3602289_bin_51), and this genome was recovered when the reads were included in this mock community.

### Description of circularized bacterial genomes

**Fig 2:**
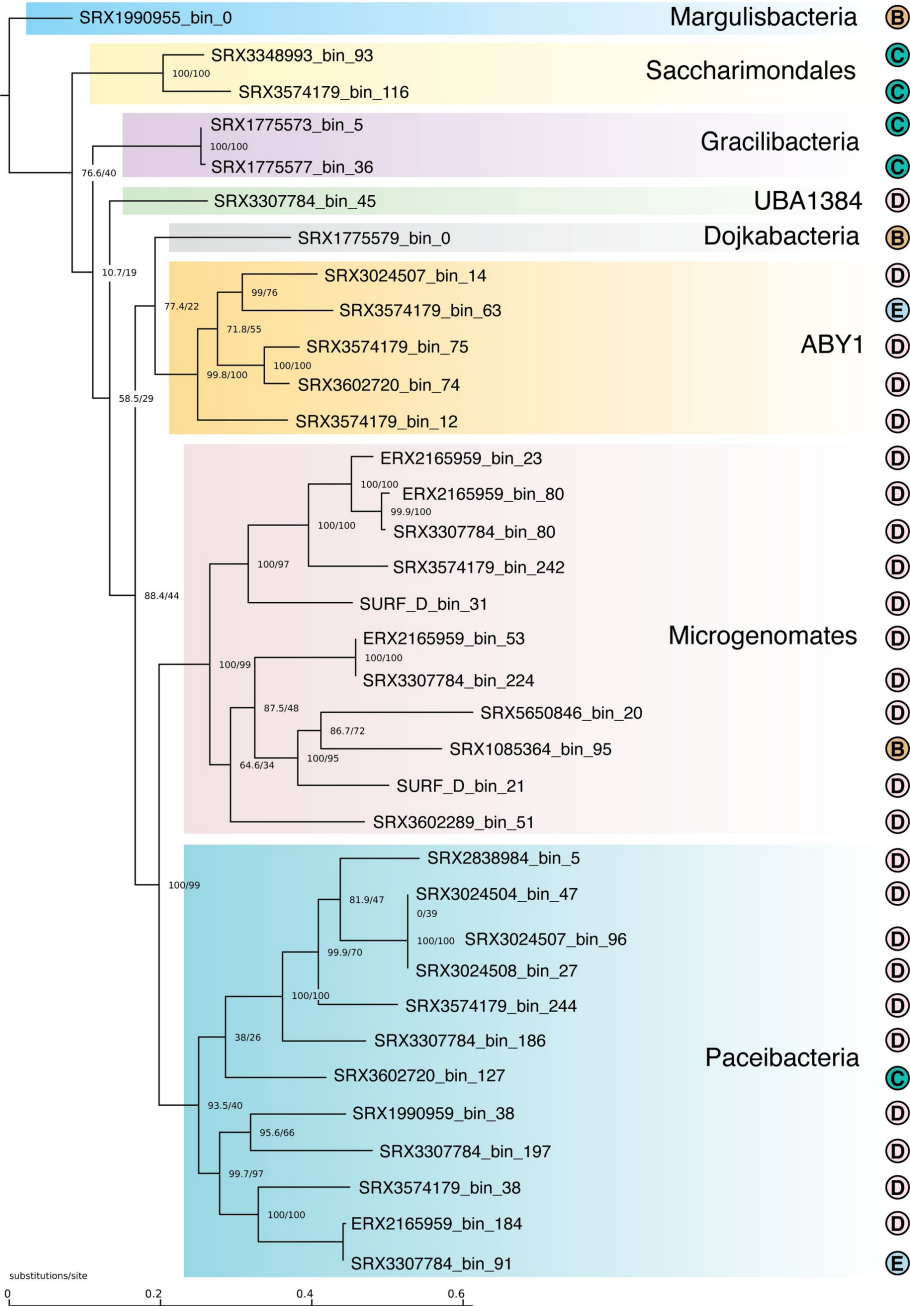
CPR Phylogenetic Tree based on SSU structural alignment. At the base of the branches, in the fraction the top value is SH-aLRT which is a branch test [52] and the bottom value is the bootstrap value. Class is listed for the CPR based on GTDB-TK taxonomy of that genome. The class UBA1384 is also known as Berkelbacteria. We specified the Margulisbacteria as the outgroup when creating the tree using IQ-TREE with ultrafast bootstrap [53]. The ribosomal operon structure is indicated on the right, with the letters matching the operon types as described in Fig 3.

Using our method, we circularized 34 CPR genomes, one Chloroflexi genome, and one Margulisbacteria genome (Table 2 and Fig 2). To create a phylogenetic tree, we used a structural alignment 16S rRNA genes. During this process we found that many of the 16S included large introns with LAGLIDADG homing endonucleases, an observation that has been noted in other CPR studies [21].

In general, these genomes are novel, but in one case, SRX1085364_bin_95, we found that the genome is 100% identical to a previously circularized genome (INSDC Accession CP011214.1) from that dataset [21]. Assembling the same genome as another group helps validate our findings and that with careful manual curation two different groups can come to the same assembly result despite differences with assemblers. Four of the other genomes had 16S genes that had 100% hits in NCBI. Some of the 16S genes only had percent similarity in the low 80s to other sequences in Genbank.

We compared the genome sizes before and after circularization, and in most cases the size of the genome decreased after circularization compared to the original bin. Typically, the genome shrank from a few hundred bases to a few thousand, but in some cases the genome shrank by more than 130kbp (Table 2). This shrinkage may be a result of SPAdes artifacts that MIRA determines to be lacking in read coherence. We are also aware of cases where SPAdes generates contigs that are effectively duplicates of each other apart from short stretches at the ends, and MIRA is able to resolve these into one contig. We examined cases that shrank more than 10kb and found that in nearly all of these cases the shrinkage was a result of the removal of contaminating contigs during the circularization process. This suggests that Jorg can help indicate or remove contaminating contigs in a bin. In one case, approximately 24Kb of one contig was misassembled and that portion of the contig did not appear in the final circularized genome. This is a minor instance of what we call “Chaos” which we describe further in the next section.

### Misassembled contigs can be found with MIRA, *i*.*e*. Chaos

In approximately 10–20% of our attempts to reassemble a bin with MIRA, we end up with many more short contigs than what was in the original bin. SRX3024505_bin_48 started with just 7 contigs with coverage 21X and a GC content of 59%. Superficially, it looks like a reasonable bin. GTDB-Tk classifies it as a CPR in the Gracilibacteria class. However, after going through 5 rounds of our method, we ended up with 136 contigs, *i*.*e*., this a Chaos bin.

We do not know exactly what happened in the case of SRX3024505_bin_48, but we see Chaos routinely during reassembly with MIRA. In some cases, we have been able to conclude that Chaos results from insufficient read support for the largest k-mer used in the original SPAdes assembly. Put differently, the assembly graph wasn’t sufficiently well connected at the highest k-mer used. To determine if the Chaos of SRX3024505_bin_48 was solely a result of using MIRA, we used the same reads that we gave to MIRA as input into a SPAdes assembly. We ended up with 47 contigs, which was still significantly worse than the original bin. To visualize the assembly graph, we redid the assembly using Unicycler [54], which leverages the results from SPAdes. The assembly graph shows no connections between the contigs, further suggesting that there was a lack of read coherence in the original assembly (S3 Fig). It is worth noting that the size of SRX3024505_bin_48 remained relatively constant during the testing and reassembly process.

In some cases, Chaos can occur on portions of contigs. For SRX3574179_bin_75, the final genome is 24,499bp smaller than the original bin. Further investigation revealed that an approximately 24Kb portion of one of the contigs from the original bin did not make it into the final genome. We mapped the reads back to the original bin and assembled these reads with Unicycler. This produced nearly the final genome but also shattered the 24Kb portion of one of the contigs into 29 additional contigs that have no connections (S4 Fig). We do not know how common it is to have Chaos portions of contigs, but reassembly of only the reads mapping to the original bin, as we did here, can help reveal these issues.

Chaos predominantly occurs when the coverage is less than ~30X. Most of the genomes we successfully circularized have much higher coverage. Based on our experience, we believe that coverage requirements for successful circularization of genomes from metagenomes are significantly higher than for isolates. Although Chaos is a disappointing result in assembly, knowing that a bin likely has misassembled contigs is valuable.

### CPR appear to have a clade-specific pattern of unlinked ribosomal operons

**Fig 3:**
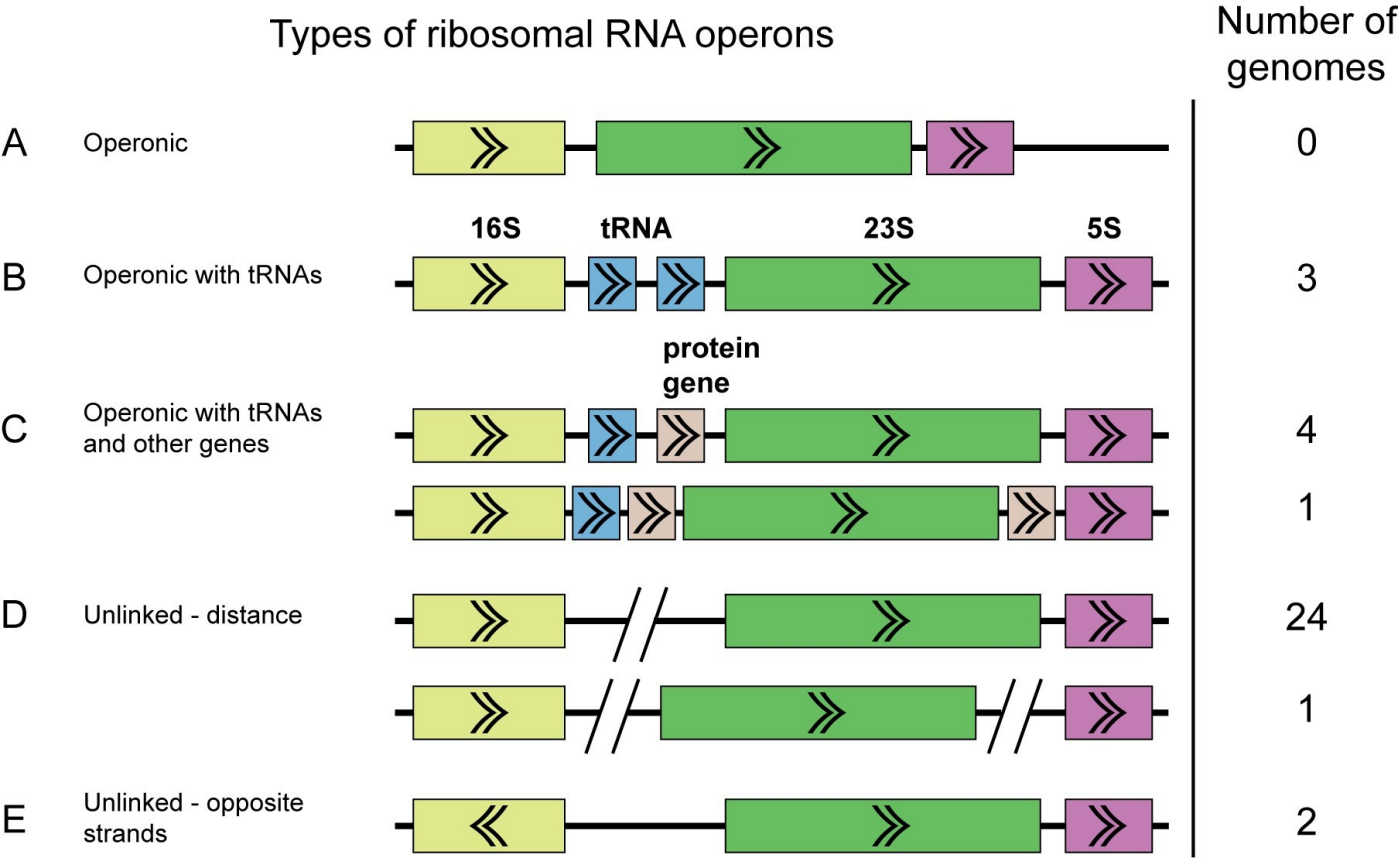
Diagram of placement types of ribosomal RNA genes. Number of genomes in this study for each category are indicated in the rightmost column. (A) Operonic. The 16S (yellow), 23S (green), and 5S (purple) ribosomal RNA genes are in an operon. (B) Operonic with tRNAs (blue). The three ribosomal RNA genes are still in an operon, but one or more tRNAs are located in the spacer between the 16S and 23S genes. (C) Operonic with tRNAs and protein coding genes (beige). The three ribosomal RNA genes are still in an operon, but one or more tRNAs or protein coding genes are located in the spacer between the 16S and 23S genes or 23S and 5S genes. It is not unusual to find that the protein coding gene is a homing endonuclease. (D) Unlinked ribosomal RNA genes by distance. The 16S gene is unlinked from the 23S and 5S genes, or the 23S is also unlinked from the 5S gene, by enough distance (>2000bp) and intervening genes on the opposite strand where it is not possible for them to be transcribed from the same promoter. (E) Unlinked ribosomal RNA genes that are on opposite strands. The 16S is on the opposite strand from the 23S and 5S genes.

Typically in bacteria and archaea, the 16S, 23S, and 5S ribosomal RNA genes are found in an operon in the order 16S-23S-5S [55] (Fig 3A). In contrast, we noted that in the CPR genomes that we circularized, nearly 80% of them (27/34) had unlinked 16S and 23S genes and sometimes unlinked 23S and 5S genes (S1 Table). Notably, the two Saccharimondial and two Gracilibacterial genomes had operonic ribosomal RNAs, while 27 of the remaining 30 CPR genomes had unlinked ribosomal RNA genes, suggesting that the presence of unlinked ribosomal operons may be clade-specific within the CPR phylogeny (Fig 2).

**Fig 4:**
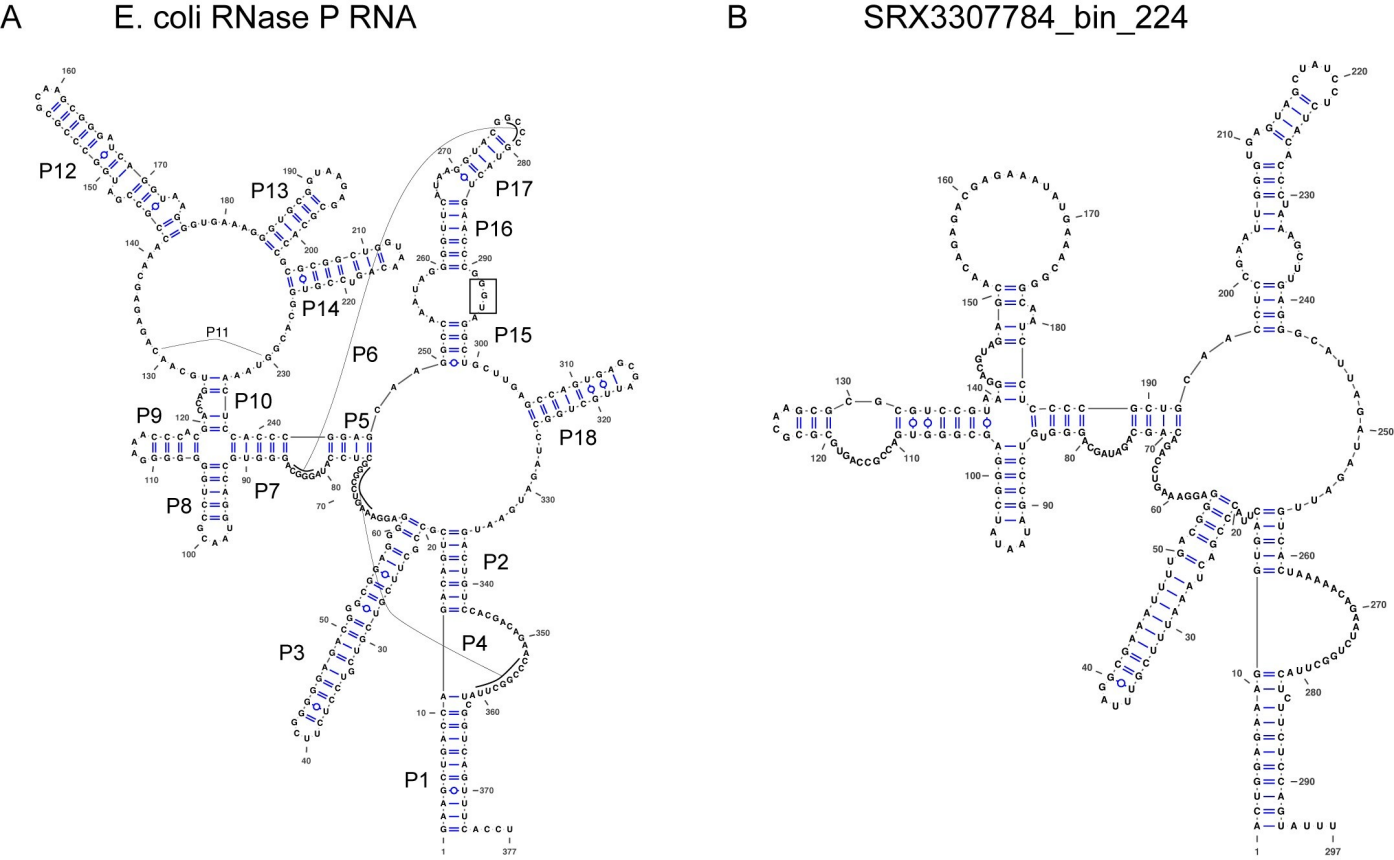
RNase P RNA can have diverged forms in CPR genomes. Structures were drawn using VARNA (Visualization Applet for RNA, http://varna.lri.fr/) [67]. (A) Structure of RNase P RNA from *Escherichia coli K-12* substr. MG1655. Helices P1-P18 are labeled. The “UGG” sequence in the P15 loop that binds to the 3’ end of the pre-tRNAs is highlighted by a box. (B) Putative Structure of RNase P RNA from SRX3307784_bin_224 genome. Note that the P12, P13, P14, and P18 helices are missing, as well as the UGG motif. Although it is not uncommon for P13, P14, and P18 to be missing in various bacteria, it is unusual that P12 is missing. To compare the two structures, large regions of the RNA had to be refolded manually from the original cmsearch prediction.

We observed the following types of ribosomal operons in our circularized genomes: (1) operonic, but the 16S and 23S are separated by tRNAs on the same strand (Fig 3B), (2) operonic, but the 16S and 23S (or 23S and 5S) are separated by tRNAs and/or protein coding genes on the same strand (Fig 3C), (3) unlinked by distance, all three ribosomal rRNA genes are on the same strand but the 16S is separated from the 23S-5S or all three are separated by more than 2000bp and there are no possible intervening genes in the spacer regions that could connect the ribosomal genes in an operon (Fig 3D), and (4) unlinked because the 16S is on the opposite strand from the 23S and 5S (Fig 3E). In three cases, tRNA genes and/or protein coding genes on the same strand were located between the 16S and 23S or between the 23S and 5S, but there are 300-500bp regions between the genes, so in these cases the ribosomal genes may be uncoupled, but conservatively we counted them as operonic. In-depth analysis of gene spacing in operons of these genomes would be required to determine if these cases are operonic or not. In the SRX1085364_bin_95 genome, we noted that there is a homing endonuclease between both the 16S and 23S, and the 23S and 5S, creating large distances between the ribosomal RNA genes. For some genomes, the distance between unlinked 16S and 23S rRNA genes can be hundreds of thousands of base pairs, indicating that their ribosomal RNA genes can be considered unambiguously unlinked without additional analysis (S1 Table). In about half of the genomes, the distance between the 16S and 5S was smaller than the distance between the 16S and 23S. Given that our genomes span a large part of the CPR phylogeny, we infer that many of the CPR likely have unlinked ribosomal operons.

The most common type of bacterial rRNA operons are those where 16S-23S-5S are transcribed together (Fig 3A), sometimes with tRNAs between the 16S and 23S (Fig 3B), so it is notable that most of the genomes in this study have unlinked rRNA operons. Instances where 16S and 23S are decoupled are unusual, although not unknown [55,56]. Separation of 23S and 5S is very unusual in bacteria but typical in archaea [56]. Decoupling between the 16S and 23S is known to occur especially in bacteria and archaea with reduced genomes (<2Mb) [55] such as *Mycoplasma gallisepticum* [57], *Borrelia burgdorferi* [58], *Ferroplasma acidarmanus* [59], as well as obligate symbionts with small genomes such as *Buchnera aphidicola* [60], *Wolbachia pipientis* [61], and *Nanoarchaeaum equitans* [56]. The Chloroflexi genome that we circularized also has an unlinked ribosomal operon, which also supports this theory. In a recent study of isolate genomes and pairing long reads with metagenomics data, others have also noted that a large percentage of the CPR likely have unlinked ribosomal operons based on analyzing the distance between the 16S and 23S genes [62]. However, to our knowledge, no one else has checked for tRNAs and protein coding genes comprising the operon in this type of analysis. We also do not know of other studies of CPR that have documented possible separation of 23S and 5S genes, proteins in the spacer regions between ribosomal RNA genes, and 16S and 23S on opposite strands.

### Diverged forms of RNase P RNA in CPR

RNase P is an RNA-protein endonuclease involved in the maturation of tRNAs by trimming the 5’ leader of pre-tRNAs. The RNA component of this complex is considered essential for all organisms except for species of the Aquificaceae family, which contain a protein that does not require the RNA component for tRNA trimming [33], and *Nanoarchaeum equitans*, an obligate symbiont that does not have any detectable RNase P RNA in its reduced genome, nor any detectable RNase P activity [32].

Given the otherwise ubiquitous nature of RNase P RNA, we require detection of this gene as a final quality check of assembled isolate genomes and circularized genomes. However, in the set of circularized CPR genomes in this study, we found that a significant number that lacked RNase P RNA (10/35). Absent a high degree of confidence that these are indeed circular genomes, we would not have noticed this anomaly. We suspected that the RNase P RNA gene was not being detected by the models because the genomes that lacked the gene did not fall into a specific clade and the genes that were detected still had many conserved features of RNase P RNA. To find the missing genes, we reduced the bitscore threshold below the model noise cutoffs when running cmsearch from the Infernal software package [63]. The noise cutoff is the score generally considered to be the score of the highest scoring false positive for that model (Infernal User’s Guide, https://infernal.janelia.org). After reducing the thresholds, we were able to detect the missing RNase P RNAs.

Most of the RNase P RNA genes that we found, even the ones we found initially, required extensive manual refolding because of the diverged structures with either large extensions of helices (S5 Fig) or missing helices. Many are missing P13, P14, P16, and P17, which is not unusual (See Fig 4A for helix locations). However, the RNase P RNA from SRX3307784_bin_224 appears to be missing P12 (Fig 4B), which is highly unusual because this helix is one of the most conserved across the tree of life [40], and it is only known to be missing in *Mycoplasma fermentans* [64] and members of the archaeal family Thermoproteaceae [65]. The closely related genome in this study, ERX2165959_bin_53, is also missing P12 (S6 Fig). Another unusual feature is that approximately two-thirds of the RNase P RNA (23/35) are missing the UGG motif that binds to the CCA in pre-tRNAs. This motif tends to be missing from cyanobacteria and chloroplasts, which may not have the CCA in their pre-tRNAs [66]. Given that cyanobacteria are one of the closer lineages to the CPR in the bacterial tree, the loss of the UGG motif may be related to lineage. A final example of a diverged feature is that the RNase P RNA from SRX1775579_bin_0 appears to be missing nearly the entire P15 helix (S7 Fig). This helix is responsible for establishing binding to pre-tRNAs in bacteria and typically contains the UGG motif, although it is missing from all known RNase P RNA in eukaryotes and some archaea.

Finding these diverged forms of RNase P RNA would not have been possible without having confidence that we had a complete genome. Ideally if we had been using our own datasets and still had DNA from the sample, we could generate RNA via reverse transcription and use a method like SHAPE-SEQ to experimentally confirm the RNA secondary structures [68]. Finding these diverged structures also illustrates that we may find diversity of genes in metagenomics data when we are no longer restricted by what we can culture in the laboratory.

### Detection and assembly of megaphage genomes

In the process of circularizing genomes, we circularized what we first thought were two novel isolates with small genomes (~0.5 Mb). However, since one of our standard checks is to run all circular sequences against a full set of Rfam models, these immediately stood out because the only RNAs detected were tRNAs and a tmRNA. Also, GTDB-tk was unable to assign a taxonomy. Cursory BLASTX searches of large regions of the genome yielded only distant hits. Based on this, we decided that they were likely megaplasmids, but have now concluded that they are megaphage based on a recent publication [69].

SRX3024509_bin_4 is an example of one of these putative megaphages. It is 536,059 nt long and codes for 74 tRNA sequences along with one tmRNA. We have seen more than 10 similar—in terms of size and RNA content—megaphages in a variety of environments. They appear to be quite common and if we expanded our size limits, we believe we would find many more. Our method should in theory perform even better on plasmids and viruses than on normal genomes since the former are less likely to have repeats. Given the development of viral and plasmid specific assembly tools for metagenomes [70–72], future work on Jorg can be compared to these tools or incorporate similar tactics to aid assembly of these types of DNA molecules. Extraction of plasmids and viruses from the metagenomes is a matter for future work.

## Discussion

We believe it is crucial to have a substantial collection—on the order of hundreds per phylum—of genomes that approach traditional finished genome standards as closely as possible, such as having a single circular, contiguous sequence with an error rate less than 1 per 100,000 bp [73]. Given that we have not yet succeeded in isolating many of the species found in metagenomics datasets, our focus is on extracting their genomes from environmental metagenomes and enrichments. By checking that assembled genomes are circular and possess all standard known components of genomes—such as RNAs without which life as we know it cannot exist—are present, we gain high confidence that we had nothing but the genome and that the genome was not falsely circularized. We believe that circularity is a top criterion for a high-quality assembly, along with checks for misassemblies. We see a clear need for an ongoing curation of collections of genomes. As more circularized genomes are generated from metagenomics data, comparisons will help expose misassemblies and false circularization. For metagenomics data, checking for misassemblies is crucial because they can produce chimeric genomes and lead to erroneous conclusions and information in public genomic databases [74].

To help facilitate MAG circularization, we have described a method called Jorg. This method is meant primarily for small genomes (<3Mbp) that have few repeats, but could help set the stage for bin improvement. The code for iterating to pull reads mapping to a bin and reassembly with MIRA are available on Github (https://github.com/lmlui/Jorg) and is available as a beta app in KBase [30]. During the development of our circularization method, we learned some lessons about when it is the most successful and instances where it will likely fail:

1. Our method works well for small genomes without repeats of significant length. Exact repeats longer than the fragment length remain an issue. If the fragment length is less than the length of a repeat, then it cannot resolve the repeat in the assembly. Once repeats get above the fragment length, the process will—and should—fail.
2. We noted that genomes with rRNA copy numbers greater than one will almost always fail to circularize. Binners almost always fail to correctly bin multiple copies of rRNA operons as they end up on shorter contigs with coverage that is a multiple of the single copy stretches. Because we do “digital primer walking”, it is possible to extend a contig to cover a portion of an unbinned contig containing the ribosomal RNA genes. While our method will not result in automatic circularization in this case, it can set the stage for further manual curation and possible eventual circularization.
3. Circularization of genomes from metagenomes depends heavily on coverage. All of the genomes we circularized had coverage greater than 29X (Table 2), but it may be possible to circularize a genome with lower coverage. However, in these cases, circularization will generally require manual intervention and we do not know how it would be automated.
4. Circularizing genomes with high GC content is more difficult. This is not particularly surprising given that all of this data was Illumina sequenced and there are known biases against high GC content [75].

There may be rare instances where circularity does not ensure the absence of contamination. Theoretically, it is possible to assemble a circular genome that is a composite of closely related strains. However, this would require that the strains have the same abundance in the sample and have large stretches of identical sequence to create chimeric joins. In tests of the ZymoBIONICS mock community, chimeric contigs formed between the *Salmonella enterica* and *Escherichia coli* genomes because they highly similar genomes [76], GC content (52.2% and 46.7%, respectively), and comprised the same percentage of the mock community. However, despite these chimeric contigs, we did not find any instances of contigs joining where it would be incorporated into a circular genome; the chimeric sequences were always at the ends of the contigs.

The effect of applying Jorg to high-quality bins can improve the chances of circularizing a bin, but it also depends on the genome (*i*.*e*., large number of repeats interferes with assembly by MIRA) and the composition of the metagenome. Bins that are more complete will likely have more success. For example, the *Lactobacillus fermentum* genome in the mock community was split across 3 bins by MetaBat 2 and were only slightly improved by applying Jorg (N50 increased by ~1000bp). However, combining all three bins to simulate a more complete starting bin resulted in larger improvement (N50 increased by ~10kbp, number of contigs reduced to 64 from 79) with no introduction of contamination. The effect of contamination in the initial bin is more complicated. Jorg will extend contaminating contigs, so it is important to either start with low amounts of contamination or remove contaminating contigs between iterations. However, if there are two similar strains of similar abundance in the sample, this could cause issues too. In addition to the issues with chimeras, the *Escherichia coli* and *Salmonella enterica* bins in the mock community had large increases in contamination after applying Jorg despite starting out with relatively low contamination (S3 Table). If the two genomes had not been at similar abundances, the contaminating contigs would have likely been filtered out during the application of Jorg. Taken altogether, efforts to start with high quality bins and using metabinners such as DAS-Tool [26] could help with obtaining circularized genomes using Jorg, but with awareness of the effects of contamination in the process.

We are well aware that circularity is not sufficient in and of itself to account for all of the genetic material of a microbe, *i*.*e*., multiple chromosomes, plasmids, etc. may comprise a genome and not just one chromosome. As long read technologies become more feasible for metagenomics, such as those developed by Pacific Biosciences and Oxford Nanopore Technology, assembling microbial genomes and resolving the types of issues described here will become easier [22,27]. In addition, long read technologies are starting to yield methylation patterns that can be used to associate multiple replicons with each other, so it may be possible to resolve if there are multiple chromosomes and plasmids in a genome [77].

We have begun wider application of Jorg across metagenomes and have evidence that it will be possible to circularize many more genomes. These full-length genomes can serve as scaffolds for future assemblies, improve comparative genomics and provide better markers for amplicon analysis; and facilitate comparative genomics of gene context and genome evolution. If we can link 16S genes to the functional potential that we can see in the genome, it will allow us to glean more information from 16S studies in terms of metabolic inference. As we continue to generate high quality complete genomes from metagenomics data, we will be able to more accurately analyze the functional potential of microorganisms that we cannot yet culture.

## Materials and methods

### Metagenomics datasets

We used datasets from the NCBI Sequence Read Archive (https://www.ncbi.nlm.nih.gov/sra). Accession numbers are listed in Table 1. In addition, we obtained the SURF datasets from Lily Momper [42].

### Read processing assembly

Metagenomic reads were preprocessed using BBtools version 38.60 to remove Illumina adapters, perform quality filtering and trimming, and remove PhiX174 spike-ins. We are not aware of any published papers documenting these tools. However, it is a standard tool suite developed at the Department of Energy Joint Genome Institute (JGI) and it is documented at https://jgi.doe.gov/data-and-tools/bbtools/. Processing was done in two passes. First bbduk.sh ran with parameters *ktrim = r k = 23 mink = 11 hdist = 1 ref = adapters*.*fa tbo tpe 2*. This was to remove any remaining Illumina adapters given in adapters.fa (standard Illumina adapters). Then bbduk.sh was run again with parameters *bf1 k = 27 hdist = 1 qtrim = rl trimq = 17 cardinality = t ref = phix174_Illumina*.*fa*. This was to perform quality filtering and trimming as well as remove Illumina PhiX174 spike ins given in the file phix174_Illumina.fa.

### Genome assembly and classification

Assembly was performed using SPAdes version 3.13.0 [43,44] with parameters—*meta -k 21*,*33*,*55*,*77*,*99*,*127*. Following assembly, from BWA version 0.7.17-r1188 [78], we used the BWA-MEM algorithm with default parameters to map the reads to the set of contigs produced by the assembly. We did this to obtain the BAM file required by MetaBAT 2 version 2.0 [45]. We used MetaBAT 2 with parameters—*unbinned—minContig 1500—maxEdges 500* to bin the contigs. The iterative assemblies were performed using MIRA 5.0rc1 [8]. The parameters set were *-NW*:*cac = warn*, *-CO*:*fnic = yes -AS*:*nop = 6*:*sdlpo = no -KS*:*fenn = 0*.*3*. We used Pilon version 1.23 [50] with default parameters to run final read coherence checks and clean up minor indels. Taxonomic classification was generated using GTDB-Tk version 0.3.3 [46].

We carried out all of the work using standard Haswell architectures with 20 cores and 256 GB of main memory. SPAdes is generally memory limited and that is where the high point of memory use occurred. Most of the iterative binning work is possible on a standard desktop or even a laptop with 32 GB of memory as long as the coverage of the candidate genomes doesn’t exceed ~100X.

### Gene annotation

All of the RNA annotations were generated by Infernal 1.1.2 [63] using *cmsearch* with parameters—*notextw—cut_tc*. We also used in-house scripts to handle RNA clan processing [79]. We used RFAM version 14.1 for the models except when we used SSU-ALIGN (see Phylogenetic Tree section below) which uses built-in custom models. For RNase P RNA, we reduced the required bit score threshold 5 using the bacterial Class A model (RF00010) to find the diverged forms. Gene calling was done using Prodigal version 2.6.3 [80]. We used *prodigal* with parameters *-n -p single*.

### Phylogenetic tree

A tree was constructed from a structural alignment of the 16S genes generated by SSU-ALIGN version 0.1.1 with default parameters [81,82]. Some 16S genes required manual folding and adjustments to correct the structural alignment. We used IQ-TREE version 2.0-rc1 [53] to generate the tree via the web server at Los Alamos National Laboratory.

### Mock community assembly and analysis

To understand the effects of bin improvement and bin characteristics on the success of Jorg, we applied the method to sequencing data of a mock community. We obtained Illumina sequencing data of the ZymoBIOMICS Microbial Community Standard (Zymo Research Corporation, Irvine, CA, USA. Product D6300) from the Joint Genome Institute. The Illumina reads were assembled and binned as described in the “Genome Assembly and Classification” section. For analysis, we used the reference genomes that are provided in the ZymoBIOMICS Microbial Community Standard protocol.

The ZymoBIOMICS microbial community is composed of 8 bacterial and 2 yeast species: *Pseudomonas aeruginosa* (4 SSUs), *Escherichia coli* (7 SSUs), *Salmonella enterica* (7 SSUs), *Lactobacillus fermentum* (5 SSUs), *Enterococcus faecalis* (4 SSUs), *Staphylococcus aureus* (6 SSUs), *Listeria monocytogenes* (6 SSUs), *Bacillus subtilis* (10 SSUs), *Saccharomyces cerevisiae* (109 SSUs, haploid genome), *Cryptococcus neoformans* (60 SSUs, haploid genome). Each of the bacteria are present as 12% of the community and each of the yeast at 2% of the community. For the purposes of this analysis, we did not analyze the use of Jorg on the yeast genomes. To test recovery and circularization of a CPR, we took one of the assembled genomes from this study (SRX3602289_bin_51), used mirabait to collect the reads, and added them to the Illumina read pool. We used SPAdes to assemble the Illumina reads and used MetaBat2 to bin the contigs.

## Supporting information

S1 Fig Example Coverage Graphs from Genomes Assembled in this Study. Sequencing coverage graphs of genomes ERX2165959_bin_23, ERX2165959_bin_80, SRX1775573_bin_5, SRX1775577_bin_36, and SRX1775579_bin_0. Coverage is generally even with no areas of unusually low coverage. Variation in coverage at the ends of the chromosome are read-mapping artifacts since reads that span the chromosome ends may not map to these regions.
(TIF)

S2 Fig Investigation of factors influencing computational requirements to run Jorg. Data shown is from 10 iterations of Jorg on bins generated from the ZymoBIOMICS Microbial Community Standard. Reads were assembled with SPAdes and contigs were binned using MetaBat 2. The reads were subsampled and the starting size of the interleaved reads file for baiting was 1.5Gb. Run time is dominated by the baiting process for bins with starting size of approximately 1Mbp or less. For larger bins, the run time is dominated by the assembly by MIRA.
(TIF)

S3 Fig Chaos of SRX3024505_bin_48. Assembly graph from Unicycler assembly. Visualization produced using Bandage [83].
(TIF)

S4 Fig Chaos of SRX3574179_bin_75. Visualization produced using Bandage. The two contigs comprising the circular graph mapped exactly back and represented the entire circularized genome, but one portion of an original contig shattered into 29 small contigs upon reassembly with Unicycler.
(TIF)

S5 Fig Putative RNase P RNA structure of SRX2838984_bin_5. This RNase P RNA appears to have an extended P15 helix compared to typical RNase P RNA (see the *E*. *coli* RNase P RNA structure in Fig 4A of the main text). Yellow highlights indicate the portions of the RNA that had to be refolded manually. This amount of refolding was not unusual for the RNase P RNAs found in this study.
(TIF)

S6 Fig Putative RNase P RNA structure of ERX2165959_bin_53. This structure is missing P12, P13, P14, and P18. It is not unusual to be missing these helices, except for P12 which is found in nearly all RNase P RNA structures.
(TIF)

S7 Fig Putative RNase P RNA structure of SRX1775579_bin_0. This structure appears to be missing most of the P15 helix.
(TIF)

S1 Table CPR Ribosomal Operons. Information on if the ribosomal operon in a genome is operonic or unlinked, as well as additional notes and the distance between ribosomal RNA genes.
(XLSX)

S2 Table GUNC Results on the genomes from this study. The GUNC results indicate that the circularized genomes in this study are not chimeric.
(XLSX)

S3 Table MetaBat 2 Bin Information. We applied Jorg to the bins by using a subsample of the reads because the original dataset was very large. Completeness and contamination were calculated by CheckM using the lineage workflow. For the MetaBat 2 bins, we did not apply Jorg to Bin 3 because of its small size and we did not apply Jorg to Bin 6 because it is comprised of contigs from the 2 yeast genomes (note that the GTDB-Tk classification is incorrect, likely because it does not look for eukaryotic taxonomy).
(XLSX)
