# Supplementary figures and images for: A method for achieving complete microbial genomes and improving bins from metagenomics data
Source: PLoS Comput Biol. 2021 May 7;17(5):e1008972. doi: 10.1371/journal.pcbi.1008972 (PMC8172020; doi:10.1371/journal.pcbi.1008972)

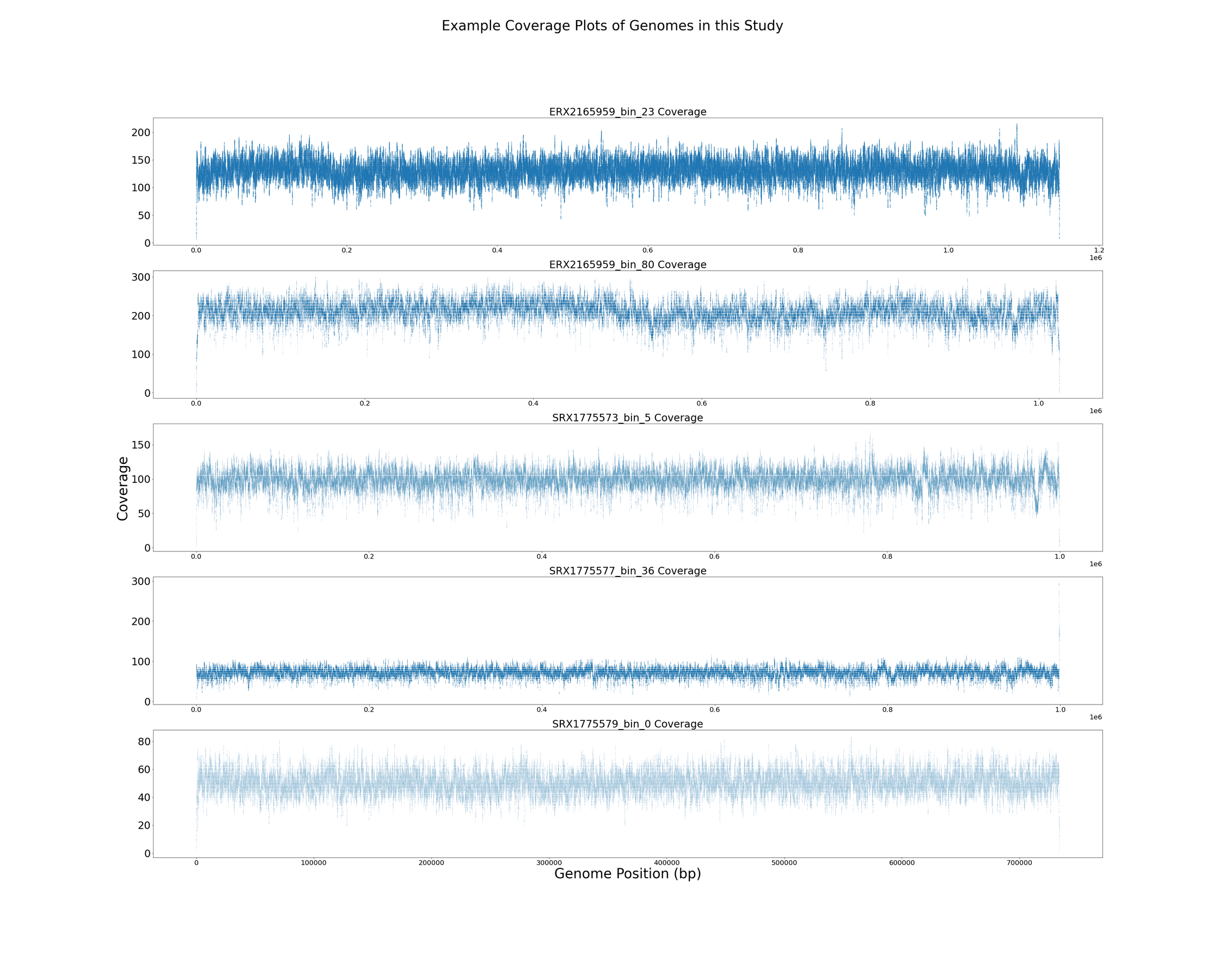

Supplement: S1 Fig — Sequencing coverage graphs of genomes ERX2165959_bin_23, ERX2165959_bin_80, SRX1775573_bin_5, SRX1775577_bin_36, and SRX1775579_bin_0. Coverage is generally even with no areas of unusually low coverage. Variation in coverage at the ends of the chromosome are read-mapping artifacts since reads that span the chromosome ends may not map to these regions. (TIF) [file pcbi.1008972.s001.tif]

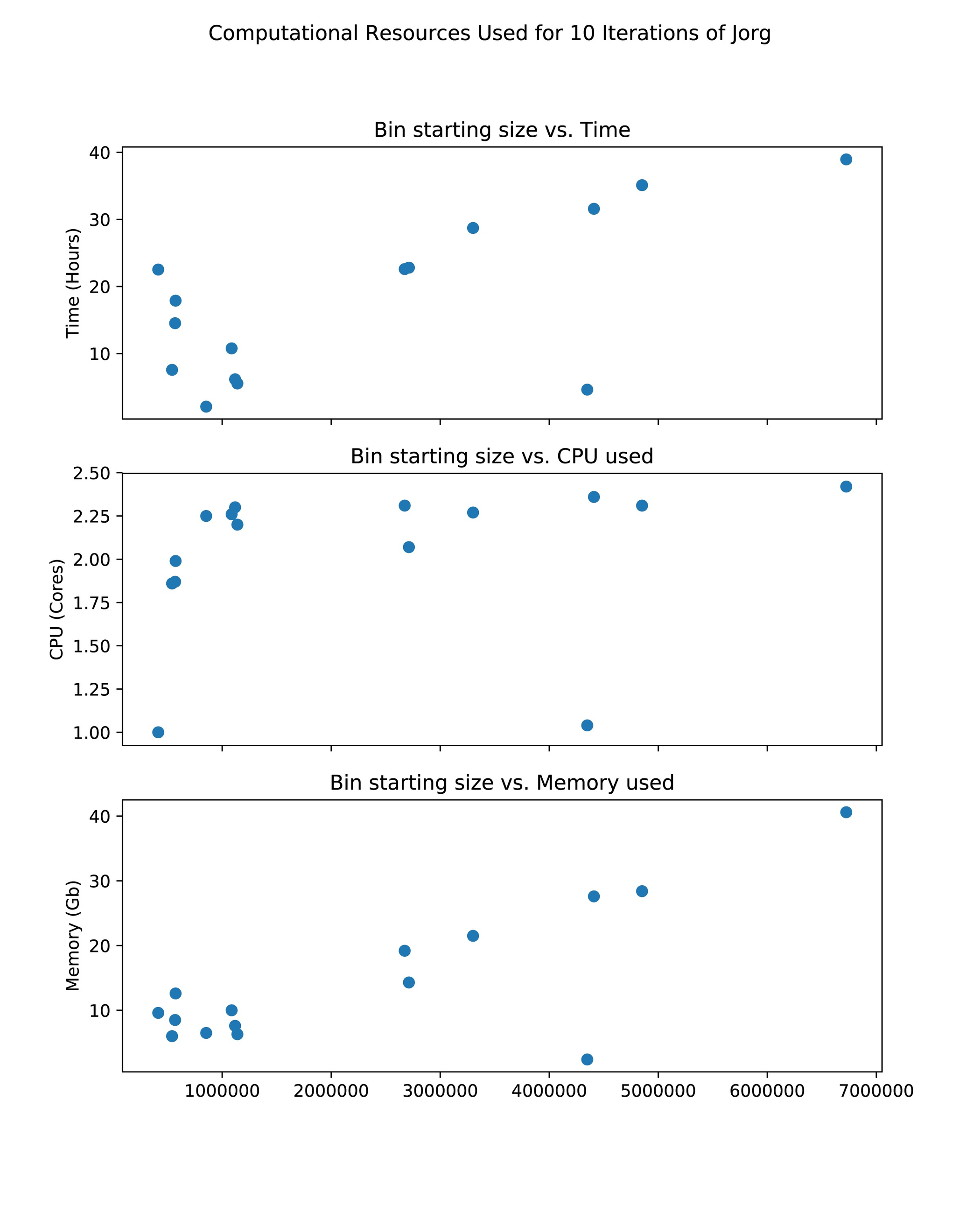

Supplement: S2 Fig — Data shown is from 10 iterations of Jorg on bins generated from the ZymoBIOMICS Microbial Community Standard. Reads were assembled with SPAdes and contigs were binned using MetaBat 2. The reads were subsampled and the starting size of the interleaved reads file for baiting was 1.5Gb. Run time is dominated by the baiting process for bins with starting size of approximately 1Mbp or less. For larger bins, the run time is dominated by the assembly by MIRA. (TIF) [file pcbi.1008972.s002.tif]

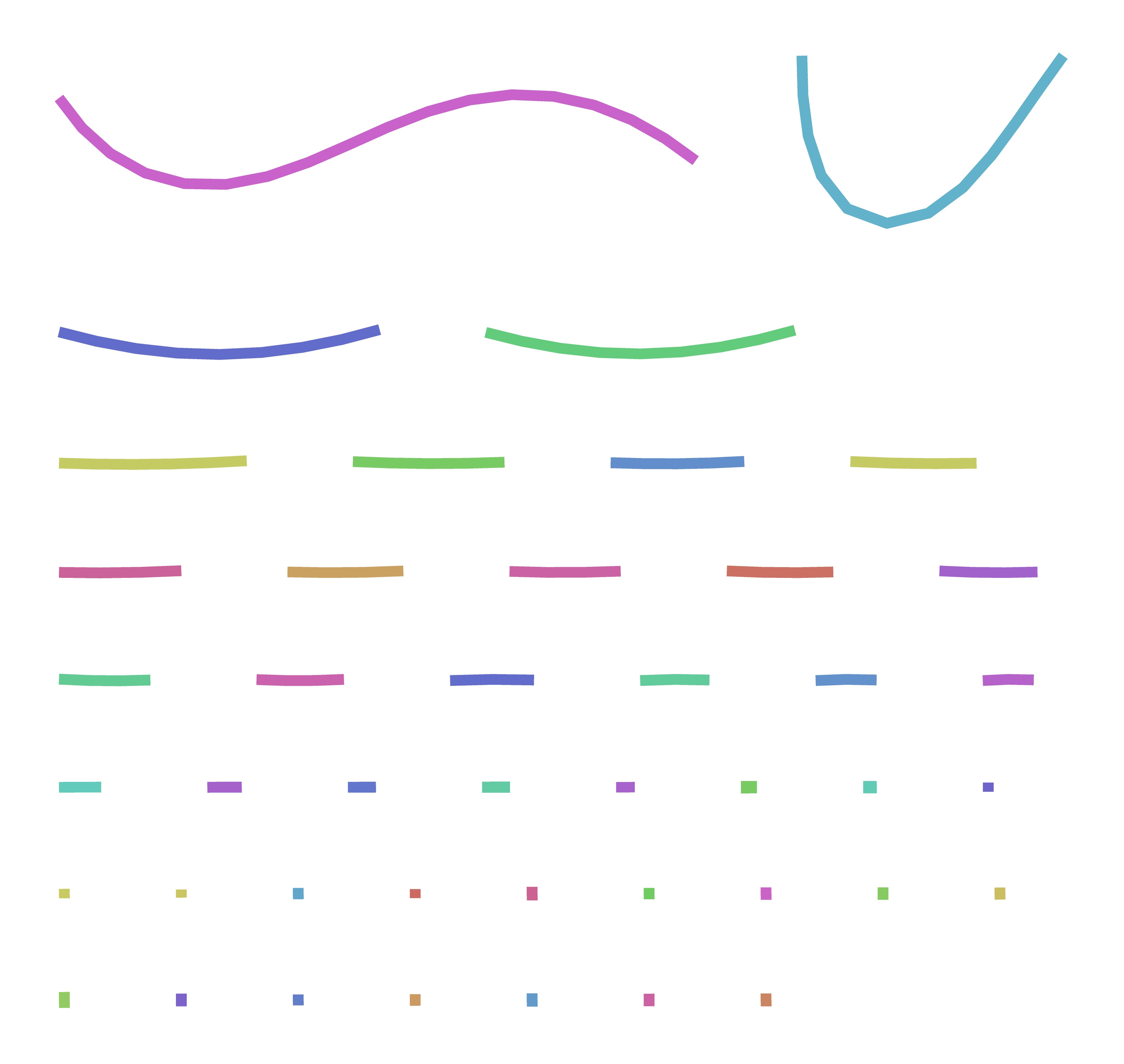

Supplement: S3 Fig — Assembly graph from Unicycler assembly. Visualization produced using Bandage [83]. (TIF) [file pcbi.1008972.s003.tif]

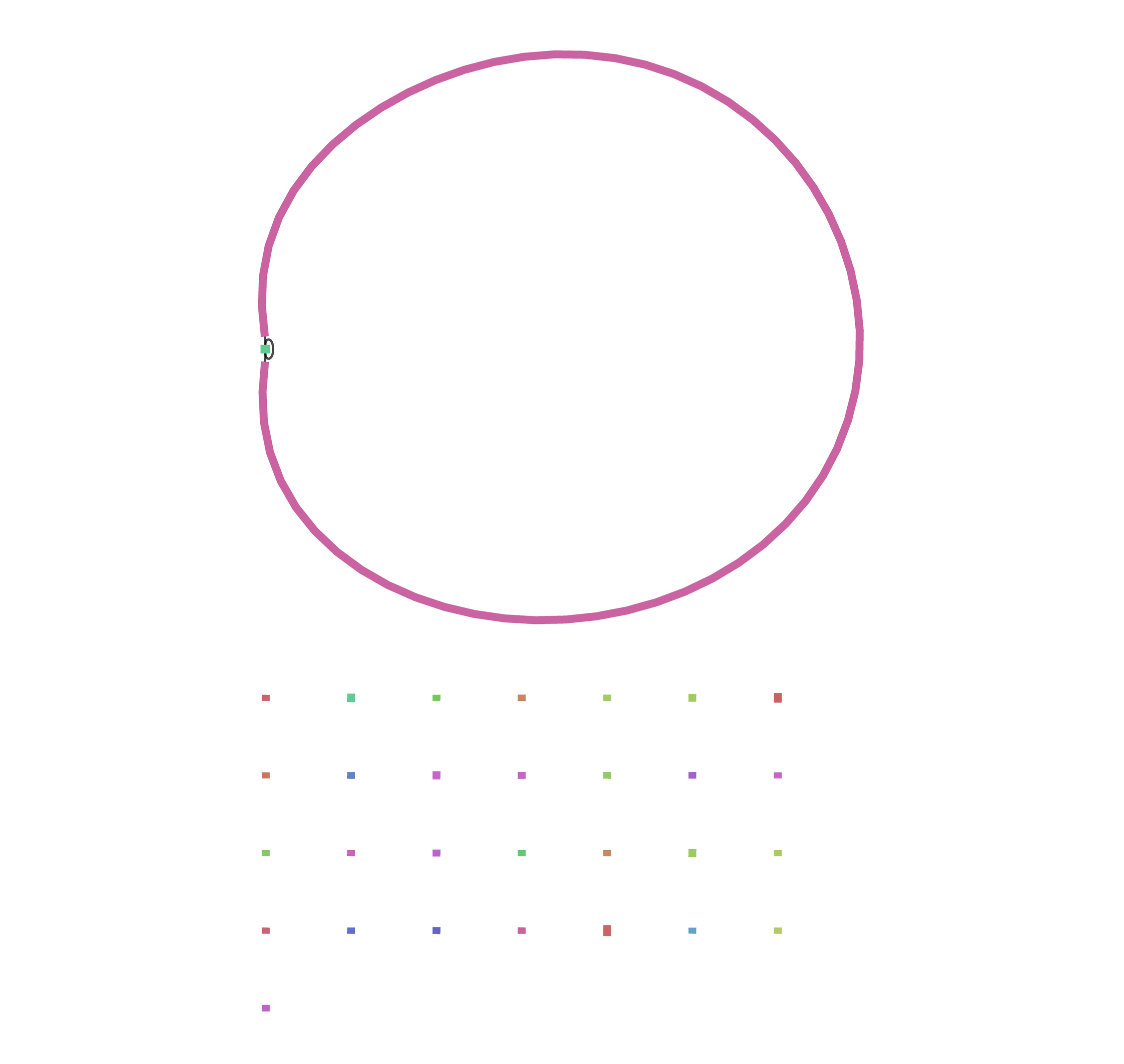

Supplement: S4 Fig — Visualization produced using Bandage. The two contigs comprising the circular graph mapped exactly back and represented the entire circularized genome, but one portion of an original contig shattered into 29 small contigs upon reassembly with Unicycler. (TIF) [file pcbi.1008972.s004.tif]

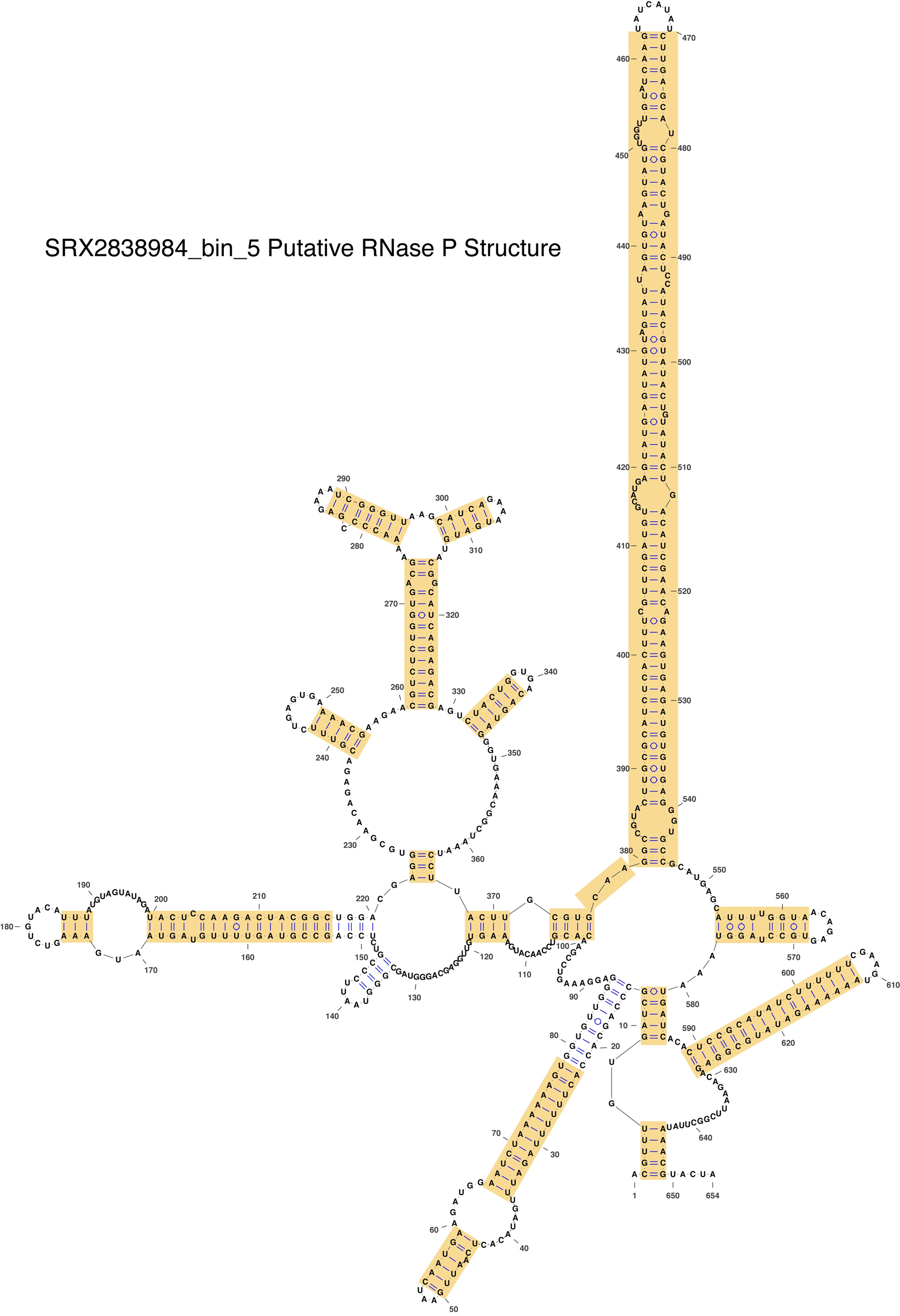

Supplement: S5 Fig — This RNase P RNA appears to have an extended P15 helix compared to typical RNase P RNA (see the E. coli RNase P RNA structure in Fig 4A of the main text). Yellow highlights indicate the portions of the RNA that had to be refolded manually. This amount of refolding was not unusual for the RNase P RNAs found in this study. (TIF) [file pcbi.1008972.s005.tif]

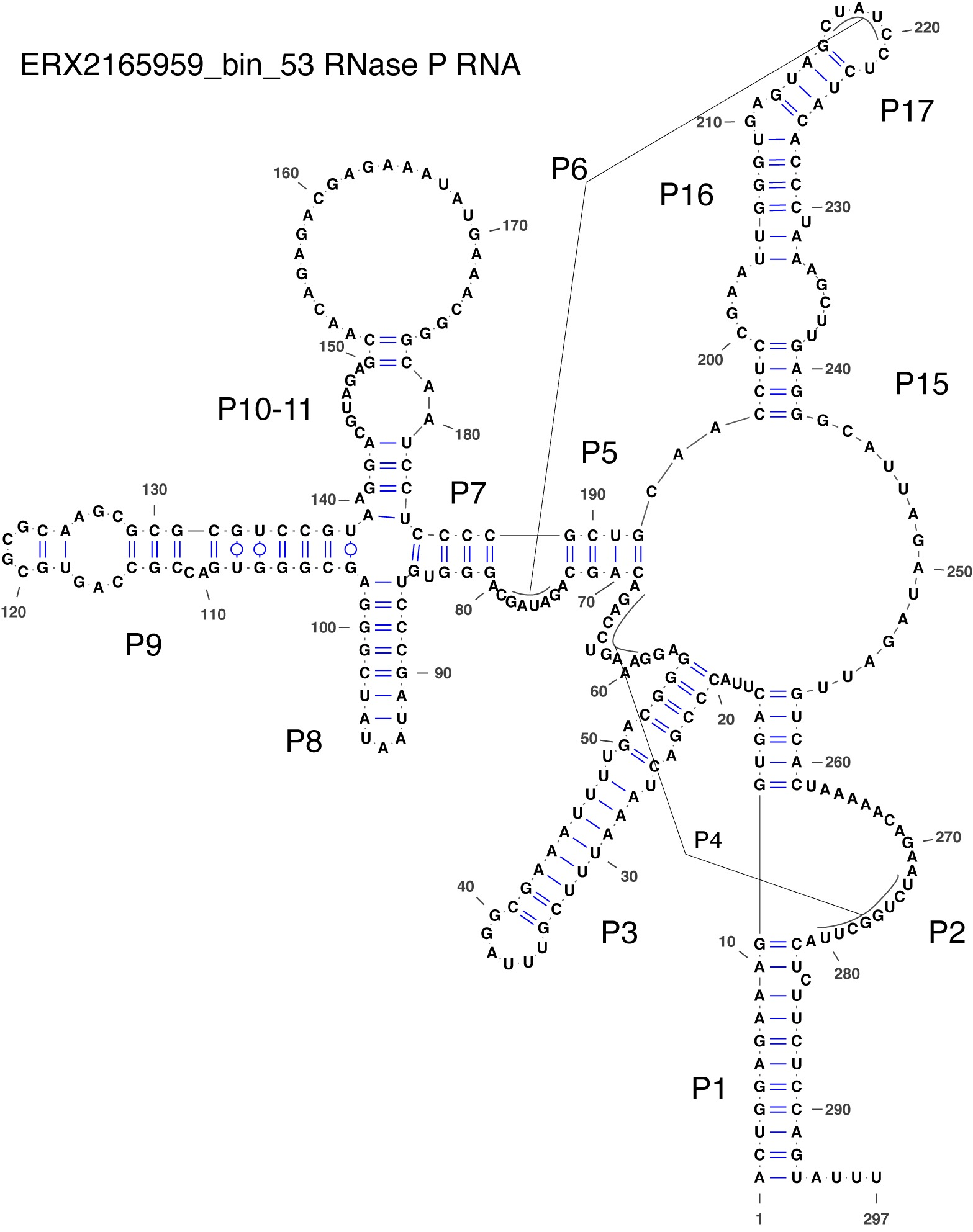

Supplement: S6 Fig — This structure is missing P12, P13, P14, and P18. It is not unusual to be missing these helices, except for P12 which is found in nearly all RNase P RNA structures. (TIF) [file pcbi.1008972.s006.tif]

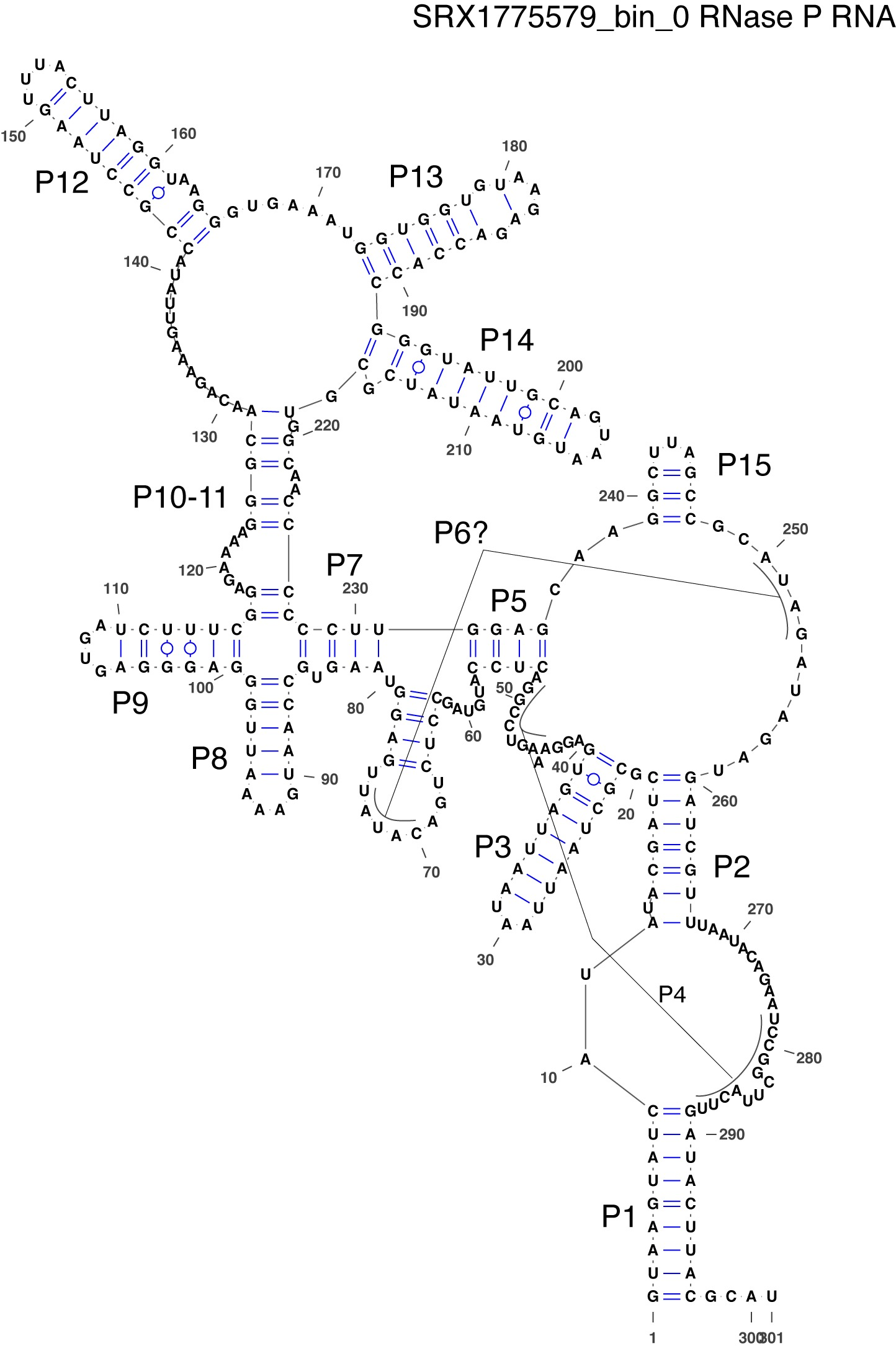

Supplement: S7 Fig — This structure appears to be missing most of the P15 helix. (TIF) [file pcbi.1008972.s007.tif]
